# Pancreatic Fibroblasts Stimulate the Motility of Pancreatic Cancer Cells through IGF1/IGF1R Signaling under Hypoxia

**DOI:** 10.1371/journal.pone.0159912

**Published:** 2016-08-03

**Authors:** Toshiki Hirakawa, Masakazu Yashiro, Yosuke Doi, Haruhito Kinoshita, Tamami Morisaki, Tatsunari Fukuoka, Tsuyoshi Hasegawa, Kenjiro Kimura, Ryosuke Amano, Kosei Hirakawa

**Affiliations:** 1 Department of Surgical Oncology, Osaka City University Graduate School of Medicine, Osaka, Japan; 2 Molecular Oncology and Therapeutics, Osaka City University Graduate School of Medicine, Osaka, Japan; Indiana University School of Medicine, UNITED STATES

## Abstract

Pancreatic ductal adenocarcinoma (PDAC) is characterized by its hypovascularity, with an extremely poor prognosis because of its highly invasive nature. PDAC proliferates with abundant stromal cells, suggesting that its invasive activity might be controlled by intercellular interactions between cancer cells and fibroblasts. Using four PDAC cell lines and two pancreas cancer-associated fibroblasts (CAFs), the expression of insulin-like growth factor-1 (IGF1) and IGF1 receptor (IGF1R) was evaluated by RT-PCR, FACScan, western blot, or ELISA. Correlation between IGF1R and the hypoxia marker carbonic anhydrase 9 (CA9) was examined by immunohistochemical staining of 120 pancreatic specimens. The effects of CAFs, IGF1, and IGF1R inhibitors on the motility of cancer cells were examined by wound-healing assay or invasion assay under normoxia (20% O_2_) and hypoxia (1% O_2_). IGF1R expression was significantly higher in RWP-1, MiaPaCa-2, and OCUP-AT cells than in Panc-1 cells. Hypoxia increased the expression level of IGF1R in RWP-1, MiaPaCa-2, and OCUP-AT cells. CA9 expression was correlated with IGF1R expression in pancreatic specimens. CAFs produced IGF1 under hypoxia, but PDAC cells did not. A conditioned medium from CAFs, which expressed αSMA, stimulated the migration and invasion ability of MiaPaCa-2, RWP-1, and OCUP-AT cells. The motility of all PDAC cells was greater under hypoxia than under normoxia. The motility-stimulating ability of CAFs was decreased by IGF1R inhibitors. These findings might suggest that pancreas CAFs stimulate the invasion activity of PDAC cells through paracrine IGF1/IGF1R signaling, especially under hypoxia. Therefore the targeting of IGF1R signaling might represent a promising therapeutic approach in IGF1R-dependent PDAC.

## Introduction

Pancreatic ductal adenocarcinoma (PDAC) is one of the most lethal types of cancer, carrying an extremely poor prognosis because it is highly invasive and shows rapid progression[1–3]. The overall 5-year survival rate remains less than 5% in all of PDAC patients[4], and ranges from 10% to 25%[5, 6] in patients who undergo curative surgery. Although the poor prognosis is due to the high invasive potential of PDAC, the molecular mechanisms responsible for the invasion activity remains unclear. PDAC is characterized by infiltrating cancer cells with abundant stromal cells[7, 8], which suggests a close interaction between the stromal cells and tumor cells. Increasing evidence indicates that the interactions between cancer cells and surrounding stromal fibroblasts play a critical role in invasion and metastasis of solid tumors[9, 10]. Studies on PDAC have revealed that mesenchymal cells secrete many cytokines such as insulin-like growth factor-1 (IGF1), hepatocyte growth factor[11], and transforming growth factor-β[12], which have an impact on disease prognosis[13, 14]. In pancreatic stromal cells, cancer-associated fibroblasts (CAFs) or myofibroblast-like cells are of particular interest with regard to PDAC microenvironment[13, 15]. Hypoxia is a common feature of various solid tumors due to their disorganized vascular system[16]. Pancreatic cancers, in particular, are clinically and histologically characterized as hypovascular tumors [17], however, little is known about the interaction between PDAC cells and stromal cells under hypoxic microenvironment[18].

Previous studies have revealed an association between progression of PDAC and overexpression of several growth factor receptors[19–21]. Our previous study by immunohistochemical study found that overexpression of insulin-like growth factor-I receptor (IGF1R) is associated with poor prognosis in patients with PDAC[22]. In addition, previous investigations have suggested a role of IGF1 in the intricate relationship between PDAC cells and stromal cells. However, no information is available regarding the significance of IGF1R in hypoxic PDAC lesions. IGF/ IGF1R signaling stimulates tumor progression in some types of cancer[23], suggesting that this system is an attractive therapeutic target. In fact, several antibodies and small-molecule kinase inhibitors targeting IGF1R are currently under preclinical and clinical development[24, 25]. Because little is known about the complex interaction between the tumor cell and its surrounding environment[26], the purpose of this study was to evaluate (1) the effect of pancreas fibroblasts on the invasive activity of PDAC cells under hypoxia; and (2) the therapeutic efficacy of IGF1R signaling inhibitor against invasion by PDAC with regard to the tumor–stromal interaction under hypoxia.

## Methods

### Cell Lines

Four pancreatic carcinoma cell lines, MiaPaCa-2, RWP-1, OCUP-AT, and Panc-1, were used. MiaPaCa-2, RWP-1, and Panc-1 were provided from JCRB Cell Bank (Osaka, Japan), and OCUP-AT was established at our department[27]. Each cancer cell line was cultured in 5% CO_2_ and 95% air. The culture medium was Dulbecco's modified Eagle medium (DMEM; Wako, Osaka, Japan) with 10% fetal bovine serum (FBS; Nichirei, Tokyo, Japan), and 0.5 mM sodium pyruvate (Sigma-Aldrich, Steinheim, Germany). Two human pancreas CAF cell lines, pCaF-1 and pCaF-2, were respectively isolated from the tumoral pancreas specimens of two PDAC patients. The pathological diagnoses in both patients were classified as invasive ductal carcinoma. The CAFs were used in the third through twelfth passage in culture. The primary culture was initiated as follows: the primary tumor was minced with scissors, and was cultivated in the culture medium. When the experimental study was performed, cells were cultured at 37°C in 20% O_2_ (normoxia) or 1% O_2_ (hypoxia). Hypoxic conditions were maintained using a modular incubator chamber (Hirasawa, Tokyo, Japan) with 5% CO_2_ and 1% O_2_ balanced with N_2_ gas. This study was approved by the Osaka City University ethics committee. Informed consent was obtained from the patients prior to the study.

### Growth factor and Compounds

IGF-1 (Sigma, St Louis, MO), anti IGF1R-neutralizing antibody (ab16890, Abcam, Cambridge, MA), and a small-synthetic phosphorylation inhibitors, picropodophyllin (PPP: IGF1R inhibitor, Calbiochem, Darmstadt, Germany) were used.

### Preparation of conditioned medium

We prepared conditioned medium (CM) from CAFs and pancreas cancer cells by seeding into 100 mm plastic dishes with 10 mL DMEM containing 10% FCS and then incubating for 3 days. To obtain CM, CAFs were washed with PBS and then incubated for an additional 3 days in 3 ml DMEM without FCS. Next, CM was collected from each dish. The supernatant was stored as CM at -20°C until use. All experiments were performed in medium contain 2% FCS. As a control, DMEM was used instead of CM.

### Reverse-transcription polymerase chain reaction (RT-PCR)

Real-time RT-PCR was done on the ABI Prism 7000 (Applied Biosystems, Foster City, CA) using the commercially available gene expression assay for *IGF1R*(Hs00609566) and *IGF1* (Hs01547656). Total cellular RNA was extracted from 4 pancreatic carcinoma cell lines with Trizol (Life Technologies, Gaithersburg, MD). As internal standard to normalize mRNA levels, amplification of *glyceraldehyde-3-phosphate dehydrogenase (GAPDH)* was used. The threshold cycle (Ct) values were used for calculation of the relative expression ratios using the formula described by Pfaffl[28].

### Flow cytometric analysis

The expression of IGF1R on PDAC cells was examined using a FACScan (BD LSR II; Becton Dickinson, San Diego, CA). Cells (2 x 10^6^ cells/mL) were fixed with 2% paraformaldehyde and incubated in PBS with anti IGF1R antibody (ab16890, Abcam) or mouse IgG1- isotype control (ab91353, Abcam) for 30 min at 22°C. Cells were subsequently labeled with FITC-conjugated secondary antibody (1:500; ab96879, Abcam) for 30 minutes at 22°C. The percentage of positive cells were calculated and compared with isotype-matched control-stained cells.

### Western blot analysis

For examining the effect of hypoxia on IGF1R expression, pancreas cancer cells were lysed 48 hours after incubation under normoxic or hypoxic conditions. Total protein (50 μg) was separated on polyacrylamide gels and transferred to polyvinylidene difluoride membranes (Millipore, Billerica, MA) with subsequent antibody (anti-IGF1R antibody; ab16890, Abcam, or anti-β-actin antibody; Cell Signaling Tec, Danvers, CO.), incubation. The bands were detected using an enhanced chemiluminescence system (Wako). Densitometry quantification was performed using ImageQuant software (Molecular Dynamics, Sunnyvale, CA) on a LAS 4000-mini Image Reader (GE Healthcare UK Ltd, Little Chalfont, UK).

### Enzyme-Linked Immunosorbent Assay (ELISA)

IGF1 in conditioned medium was quantified using IGF1 ELISA kit (R&D Systems). The cell number was counted when the CM was collected, and ELISA data was normalized by 1x10^6^ cells in all cell lines.

### Immunohistochemical staining of primary pancreatic tumors

A total of 120 patients who had undergone resection of a primary pancreatic tumor were included. The pathologic diagnoses were made according to the UICC Classification of Malignant Tumors[29]. The study protocol conformed to the ethical guidelines of the Declaration of Helsinki (1975). Sections of paraffin-embedded tissue were prepared. Immunohistochemical staining for IGF1R and carbonic anhydrase 9 (CA9) was performed using the avidin-biotin-peroxidase complex method using primary monoclonal antibody against, IGF1R (Abcam) and CA9 (clone; M75, 1:1000, Novus Biologicals).

### Immunohistochemical determination

All slides were examined by two of the authors who were blinded to clinical data. Scores for IGF1R were given for the staining intensity and the percentage of positive cells as follows: score of 0, no staining is observed, or is observed in less than 10% of the tumor cells; score of 1+, weak staining is detected in 10% or more of the tumor cells; score of 2+, moderate staining is observed in 10% or more of the tumor cells; and score of 3+, strong staining is observed in 10% or more of the tumor cells. CA-9 immunostaining was scored as 0 for 0%, 1+ for 1–20%, 2+ for 21–50%, and 3+ for >50%, Scores of 0 and 1+ were considered to be negative.

### αSMA expression of cancer-associated fibroblasts

CAFs were incubated into Lab-Tek II Chamber Slide System (Nunc, Naperville, IL, USA) for 3 days. After washing with PBS, CAFs were incubated with anti-alpha Smooth Muscle Actin (αSMA) antibody (Dako, Cambridge, UK) and with biotinylated rabbit anti-mouse immunoglobulin G (Nichirei Corporation, Tokyo, Japan), treated with streptavidin-peroxidase reagent (Nichirei Corporation, Tokyo, Japan), and countered with Mayer’s hematoxylin.

### Wound healing assay

Pancreas cancer cells were plated on 96-well plates (Essen ImageLock, Essen Instruments, Birmingham, UK) and a single wound per well was scratched with wound scratcher (Wound Maker, Essen BioScience, MI, USA). Compounds and appropriate controls were added after wound scratching and wound confluence was monitored with Incucyte Live-Cell Imaging System and software (Essen Instruments). Each pancreatic carcinoma cell lines were resuspended to a final concentration of 1.0x10^5^ cells/ml in DMEM with 2% FBS. One hundred microliters of cancer cell suspension and 100 μl of DMEM with 2% FBS with IGF1 (10 ng/ml), and CM in the absence or presence of IGF1R antibody (2 μg/ml) and PPP (0.25 μM) were added. Wound closure was observed every 3 hours for 24 h. Wound closure was determined as a percentage of wound confluence. The mean of 4 fields was calculated as the sample value.

### Invasion assay

The in vitro invasiveness was measured by two-chamber matrigel invasion assay, as previously reported[30]. We used the chemotaxis chambers with a 8 μm-pore membrane filter (Kubota, Osaka, Japan) coated with 50 μg of matrigel in a 24-well culture plate. Pancreas cancer cells (2x10^3^ cells/chamber) were seeded in upper chambers, and CM in the absence or presence of IGF1R antibody (2 μg/ml) and PPP (0.25 μM) were added. After 24 h incubation, cancer cells that invaded through a filter coated with matrigel to the lower surface of the membrane were manually counted under a microscope.

### Statistical analysis

Data were analyzed using Student's t test. The correlation between the expression level of IGF1R and CA-9 was analyzed by Spearman's rank correlation analysis using SPSS 13.0 (SPSS Inc, Chicago). A *P* value less than 0.05 was considered statistically significant.

## Results

### Effects of hypoxia on IGF1R expression in PDAC cells

**Fig 1:**
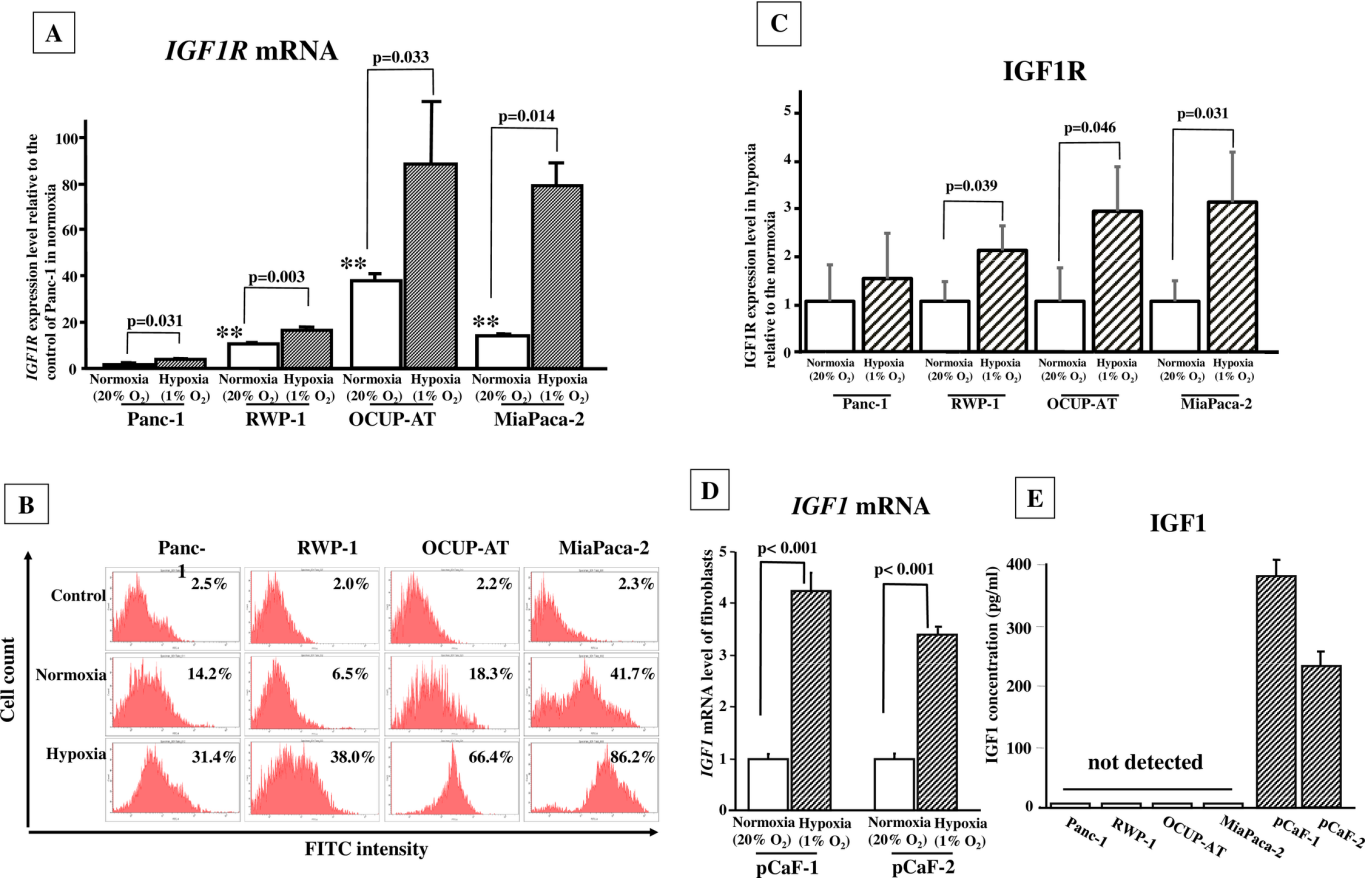
Effects of hypoxia on mRNA expression of *IGF1R* and *IGF1*, and production of IGF1 in cancer cells. (A) *IGF1R* mRNA expression was significantly higher in RWP-1, MiaPaCa-2, and OCUP-AT cells than in Panc-1 cells. The mRNA expression level of IGF1R was increased under hypoxia in all four cancer cell lines. (B) Cell surface expression of IGF1R by FACScan analysis. IGF1R expression level of Panc-1, RWP-1, OCUP-AT, and MiaPaCa-2 cells was higher in hypoxia than that in normoxia. (C) Western blot analysis. Three independent experiments were performed. IGF1R bands was calculated by β-actin as internal standard to normalize expression levels IGF1R level under hypoxia was high in 3 of four pancreas cancer cell lines, Panc-1, OCUP-AT, and RWP-1, in comparison with that under normoxia, while Panc-1 did not significantly increase under hypoxia. (D) IGF1 mRNA expression was significantly increased under hypoxia in both pCaF-1 and pCaF-2 cells. (E) The concentration of IGF1 in conditioned medium from pCaF-1 and pCaF-2 cells was 380 and 253 pg/ml under hypoxia. IGF1 production in PDAC cells was not detected.

The *mRNA* expression level of *IGF1R* was significantly higher in RWP-1, OCUP-AT, and MiaPaCa-2 (*p* <0.01) than in in Panc-1. The expression level of *IGF1R* in Panc-1, RWP-1, OCUP-AT, and MiaPaCa-2 significantly increased under hypoxia compared with that under normoxia **(Fig 1A).** Cell surface expression of IGF1R was assessed by FACScan and western blot analysis. In normoxia, IGF1R expression of Panc-1, RWP-1, OCUP-AT, and MiaPaCa-2 cells was 14.2%, 6.5%, 18.3%, and 41.7%, respectively. In hypoxia, IGF1R expression of Panc-1, RWP-1, OCUP-AT, and MiaPaCa-2 cells was1 31.4%, 38.0%, 64.4%, and 86.2%, respectively **(Fig 1B)**. IGF1R bands was calculated by β-actin as internal standard to normalize expression levels **(S1 Fig)**. IGF1R expression level in hypoxia were quantified relative to that in normoxia. IGF1R level of pancreas cancer cells, RWP-1, OCUP-AT, and MiaPaCa-2 cells, were significantly high in hypoxia, in compared with that in normoxia, but that of Panc-1 did not significantly increase under hypoxia **(Fig 1C)**.

### IGF1 mRNA expression and production

IGF1 expression and production in CAFs was affected by hypoxia. The expression level of IGF1 mRNA was significantly increased under hypoxia in pancreatic fibroblast (pCaF-1 and pCaF-2) cells (**Fig 1D**). IGF1 production from pCaF-1 and pCaF-2 cells under hypoxia was 381 and 253 pg/ml, respectively, whereas IGF1 concentration in PDAC cells was within the cutoff value (7 pg/ml) in Panc-1, RWP-1, OCUP-AT, and MiaPaCa-2 cells (**Fig 1E**).

### Association between expression of IGF1R and CA9 in 120 pancreatic specimens

**Fig 2:**
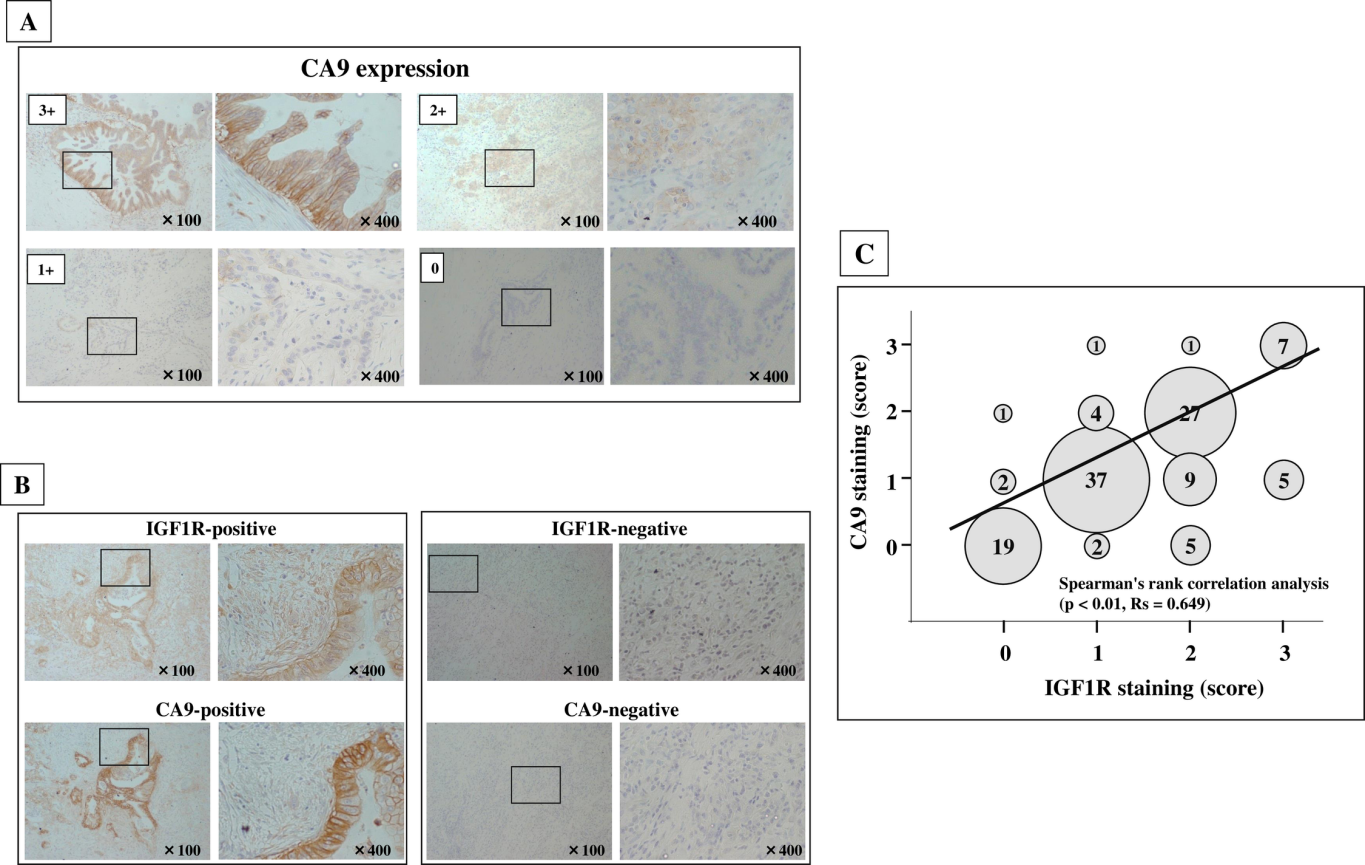
IGF1R and CA9 expression in PDAC. (A) Representative staining of CA9 cells quantified on a scoring system of 0–3 according to staining intensity (original magnification ×200). CA9 was mainly expressed in the cell membrane of PDAC cells. (B) IGF1R was mainly expressed in the cytoplasm of PDAC cells. (C) Association between IGF1Rexpression and CA9 expression in 120 pancreas cancer. A significant correlation was found between IGF1R expression level was significantly correlated with CA9 expression in PDAC cells (*p* < 0.01, *rs* = 0.649; Spearman’s rank sum test). The size of circle indicates the number of PDAC patients.

**Fig 2A and 2B shows** representative picture of CA9 staining and IGF1R staining of PDAC cells. Of 120 patients with PDAC, 41 (34%) were positive for CA9 expression and 54 (45%) were positive for IGF1R overexpression. A significant correlation was found between IGF1R and CA9 immunoreactivity in PDAC cells (*p* < 0.01, *rs* = 0.649; Spearman’s rank sum test) (**Fig 2C**).

### αSMA expression in cancer-associated fibroblasts

αSMA expression was found in both pCaF-1 and pCaF-2 cells. The percentage of αSMA-positive fibroblasts was about 60% in both pCaF1 and pCaF2 cells, respectively (**S2 Fig**).

### Effect of conditioned medium from pancreas cancer-associated fibroblasts on motility of PDAC cells

**Fig 3:**
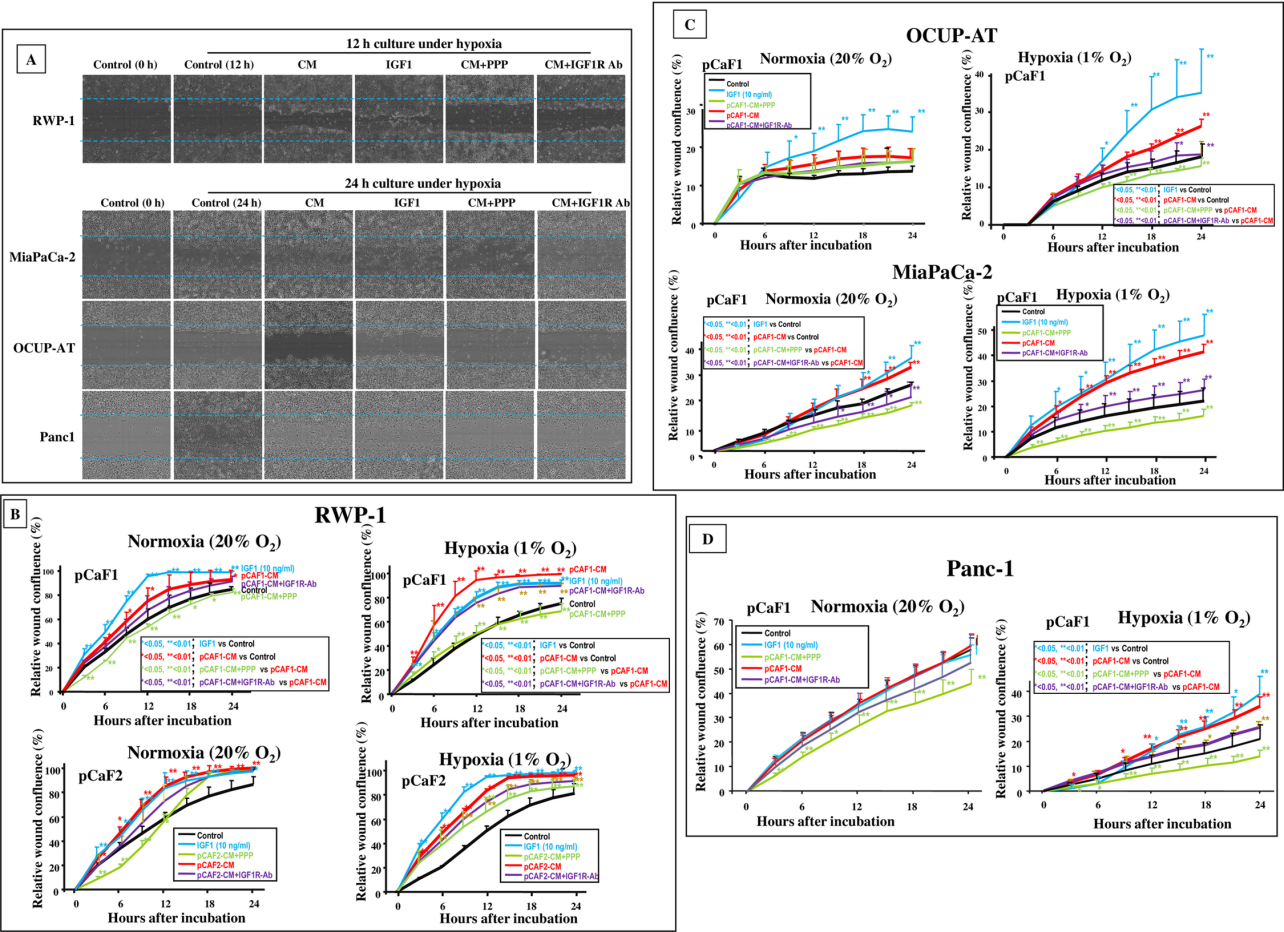
Effect of conditioned medium, IGF1, and IGF1R inhibitors on motility of PDAC cells. (A) Representative images of cell density in wound-healing assay. The number of PDAC cells migrating across the wound (broken line) was increased by conditioned medium (CM) from pancreas CAFs compared with that in control. IGF1 increased the number of migrating cancer cells. The IGF1R inhibitor picropodophyllin (PPP) and anti-IGF1R neutralizing antibody downregulated migration-stimulating activity by fibroblast CM. (B, C, D) CM from pancreas fibroblasts significantly stimulated the migratory activity of RWP-1 and MiaPaCa-2 cells, but not OCUP-AT, and Panc-1, under normoxia. Under hypoxia, the number of migrating cancer cells was significantly increased in all four cancer cell lines by CM from pancreas CAFs, and the migration-stimulating ability of CM in PDAC cells was inhibited by IGF1R inhibitors. Data are presented as mean ± SD.

**Fig 3A** shows a representative phase contrast photograph of *in vitro* wound healing assay under hypoxia. Representative pictures for the calculation of the wound confluence were described in **S3 Fig.** The motility of cancer cells was examined under both normoxia and in hypoxia. Under hypoxia, the number of migrating RWP-1, MiaPaCa-2, OCUP-AT, and Panc-1 cells was significantly increased by CM from pancreas CAFs, in comparison with the control in DMEM. Under normoxia, in contrast to that of OCUP-AT or Panc-1, the migratory activity of RWP-1 and MiaPaCa-2 cells was significantly increased by CM from pancreas CAFs (**Fig 3B, 3C and 3D**).

### Effect of IGF1 on motility of PDAC cells

IGF1 significantly increased the number of migrating RWP-1, MiaPaCa-2, OCUP-AT, and Panc-1 cells under hypoxia. Under normoxia, the migratory activity of RWP-1, OCUP-AT, and MiaPaCa-2 cells was significantly increased by IGF1, but that of Panc-1 was not (**Fig 3B, 3C and 3D**).

### Effect of signaling inhibitors on migration-stimulating activity of CM from pancreas cancer-associated fibroblasts

The migration-stimulating ability of CM was inhibited by the IGF1R inhibitor, PPP, in RWP-1, MiaPaCa-2, OCUP-AT, and Panc-1 cells under hypoxia. In contrast, PPP significantly decreased the migration-stimulating activity of CM only in MiaPaCa-2 cells under normoxia (**Fig 3B, 3C and 3D**). Under hypoxia, IGF1R-neutralizing antibody decreased the migration-stimulating activity of CM from fibroblasts in RWP-1, MiaPaCa-2, OCUP-AT, and Panc-1 cells. Under normoxia, the migration-stimulating activity of fibroblasts was decreased by anti-IGF1R antibody only in MiaPaCa-2 cells.

### Effect of signaling inhibitors on invasion-stimulating activity of CM from fibroblasts

**Fig 4:**
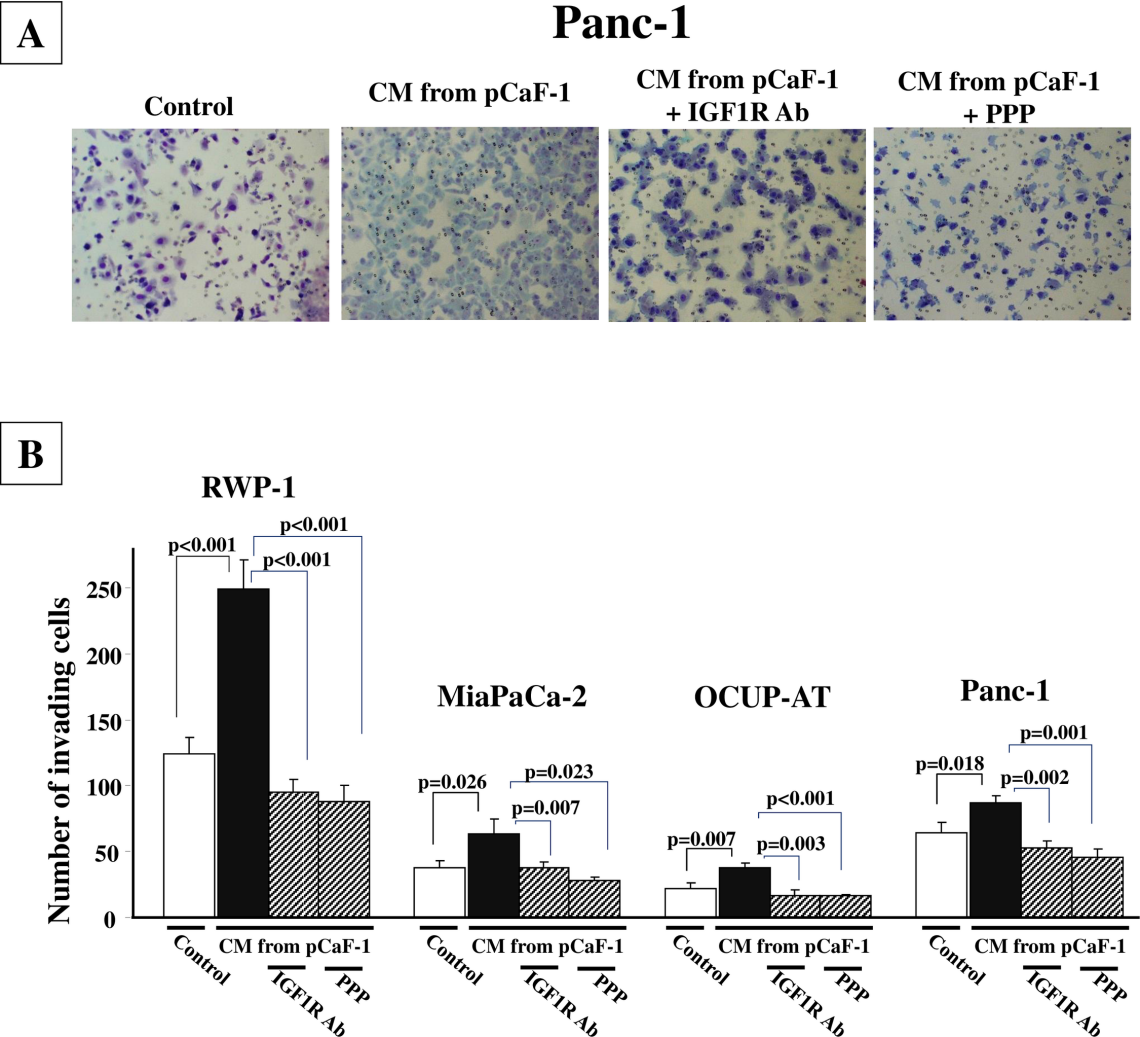
IGF1R inhibitor inhibits invasion-stimulating activity of CM from fibroblasts. (A), Representative pictures of invading pancreas cancer cells, Panc-1. The number of invaded cells into pore membrane filter was increased in the presence of CM from pCaF-1 in compared to the control. IGF1R-neutralizing antibody and PPP inhibited the invasion induced by CM. (B), CM from pCaF-1 significantly stimulated the invasive behavior of pancreas cancer cells. IGF1R inhibitor, IGF1R-neutralizing antibody and PPP, significantly inhibited the invasion seen in these cells.

**Fig 4A** is a representative phase contrast photograph of Panc-1 cells that have invaded into a 8-μm pore membrane filter. The number of invading Panc-1 cells was significantly increased in the presence of CM from pCaF-1 when compared to the control. The invasion-stimulating activity of CM was decreased in the presence of IGF1R-neutralizing antibody or PPP. CM from pCaF-1 significantly stimulated the invasion ability of all 4 pancreas cancer cell lines. IGF1R inhibitor, IGF1R-neutralizing antibody and PPP, significantly inhibited invasion-stimulating activity of CM from fibroblasts (**Fig 4B**).

## Discussion

PDAC is clinically characterized as hypovascular tumors, and these cells are continuously exposed to hypoxia. Intraoperatively Koong *et al*. measured tumor oxygenation in cases of PDAC, and revealed that a significant proportion of these cells were hypoxic[17]. Thus, PDAC cells can survive in hypoxia, as evidenced by the fact that PDAC cell lines were grown under hypoxia (1% O_2_) *in vitro* in this study. Hypoxia increased the expression level of IGF1R in PDAC cells in comparison with that under normoxia. CA9, a hypoxia-associated endogenous protein, is considered as a cellular biomarker of hypoxic regions in solid tumors[31, 32]. It has been reported that IGF1 is upregulated in PDAC tissues, but not in surrounding noncancerous tissues[33]. IGF1R is phosphorylated solely in PDAC tissues. In this histological study, the expression of CA9 was closely correlated with IGF1R expression. Using immunohistochemical investigation, we previously reported that PDAC patients with IGF1R overexpression had poor survival rates [22], suggesting that IGF1R signaling might be correlated with tumor aggressiveness in this cancer. These findings suggest that a hypoxic tumor microenvironment might affect the aggressiveness characteristics of PDAC cells via the IGF1R signaling system.

Fibroblasts have the ability to produce certain cytokines that influence neighboring cells, including malignant cells[34]. PDAC often has abundant stroma, which suggests that interactions between PDAC cells and stromal cells may play a critical role in the progression of this cancer[7, 35]. There is increasing evidence that activated fibroblasts play a pivotal role in the development of PDAC[36, 37]. In the cancer microenvironment, fibroblasts are transformed (“activated”) from their quiescent phenotype into myofibroblast-like cells which express αSMA[36, 38, 39]. In the present study, two human pancreatic CAF cell lines, pCaF-1 and pCaF-2, were established from PDAC specimens, and αSMA expression was evaluated in both these cell lines. These findings suggest that both pCaF-1 and pCaF-2 are activated fibroblasts. To clarify whether hypoxia affects cancer cell–stromal cell interaction with regard to the migratory ability of pancreatic cells, using pCaF1 and pCaF2 cell lines, we examined the effect of CM from pancreas CAFs. We observed that CM from pancreas CAFs stimulated the motility of MiaPaCa-2, RWP-1, and OCUP-AT cells under normoxia, and the migration-stimulating activity of fibroblasts on PDAC cells was greater under hypoxia than under normoxia. These findings suggest that hypoxia markedly enhances the migration-stimulating activity of pancreas CAFs on PDAC cells. We then analyzed the mechanisms responsible for this migration-stimulating activity.

Previous studies revealed that increased production of several growth factors in human stromal cells under hypoxia plays a key role in the invasiveness of cancer cells[18, 34, 40]. Pancreas CAFs produced IGF1, and its level increased under hypoxia. The invasion-stimulating ability of CM was decreased by the IGF1R inhibitor PPP and anti-IGF1R-neutralizing antibody, but not by PDGF-neutralizing antibody and TGFβ-neutralizing antibody (data not shown). IGF1 is produced from CAFs, but not from PDAC cells. These findings suggest that IGF1R signaling in PDAC cells under hypoxia may respond to IGF1 in a paracrine manner. Altogether, hypoxia increased the IGF1 expression level of pancreas CAFs, suggesting that hypoxia stimulates not only IGF1R in PDAC cells but also the IGF1 expression level of pancreas CAFs, which could result in the synergistic stimulation of migratory activity in PDAC cells in paracrine IGF1/IGF1R signaling. To our knowledge, this is the first study to find an association for IGF1/IGF1R signaling in PDAC cell-stromal cell interaction in a hypoxic cancer microenvironment.

The migration-stimulating activity of conditioned medium from fibroblasts in PDAC cells was partly inhibited by neutralizing IGF1R antibody. Not only IGF1 but also other factor(s) might affect on the hypoxia-induced migration in PDAC cells. In future studies, it will be necessary to examine other factors from fibroblasts which can affect on the migration of PDAC cells in hypoxia.

In this study, PPP and IGF1R antibody suppressed the motility of PDAC cells. Consequently, multiple phase 1–2 clinical trials testing IGF1R inhibitors in a diverse number of epithelial cancers, including PDAC, are currently ongoing. IGF1R inhibitors are available and some are undergoing phase trials[41, 42]. These findings suggest that IGF1R-targeted therapy may be useful with regard to its inhibitory effects on the motility of pancreatic tumor cells. Targeting of the pancreatic tumor hypoxic microenvironment may, therefore, represent a promising therapeutic approach in PDAC.

A high incidence of somatic K-*ras* mutations is found in some types of cancer including PDAC[43, 44]. It has been reported that K-*ras* mutation is predictive of a very poor response to signal inhibitors which inhibit the downstream effector pathways of K-*ras*, such as the mitogen-activated protein kinase (MAPK) and phosphoinositide 3-kinase (PI3K) signaling pathways in colorectal cancer[45–48]. Although all four PDAC cell lines used in this study have K-*ras* mutation at codon 12[49], the efficacy of IGF1R inhibitors against pancreatic carcinoma is independent of K-*ras* mutation status, and thus IGF1R inhibitors might be clinically useful in PDAC patients irrespective of K-ras status in PDAC patients. We observed a significantly higher expression level of IGF1R in RWP-1, MiaPaCa-2, and OCUP-AT cells than in Panc-1 cells, in which migratory activity was not increased by CM from pancreas fibroblasts. These findings indicate that aberrant IGF1R signaling may be involved in PDAC progression, and the expression level of IGF1R in PDAC may represent a predictive biomarker for response to IGF1R inhibitors. Because IGF1R represents a key molecule in the hypoxic tumor microenvironment, IGF1R inhibitors appear therapeutically promising in PDAC characterized by IGF1R overexpression.

In conclusion, we identified a motility-stimulating role of pancreas CAFs in hypoxic PDAC cells through the paracrine IGF1/IGF1R pathway. Targeting IGF1R signaling may represent a promising therapy against the progression of pancreatic carcinoma.

## Supporting Information

S1 Fig Western blot analysis. Three western pictures from 3 independent experiments were shown.
(TIF)

S2 Fig Immunohistochemical staining for αSMA. Both pCaF1 and pCaF2 cells contain αSMA-positive cells (asterisks) and αSMA-negative cells (arrows).
(TIF)

S3 Fig Representative pictures for the wound-healing assay. Pictures shows initial wound mask at 0 hours (yellow) and wound mask at 12 or 24 hours (blue). Relative wound confluence (%) was calculated as 100 x wound closure area at each time (blue) /wound area at time 0 (yellow).
(TIF)
