# Supplementary figures and images for: Pancreatic Fibroblasts Stimulate the Motility of Pancreatic Cancer Cells through IGF1/IGF1R Signaling under Hypoxia
Source: PLoS One. 2016 Aug 3;11(8):e0159912. doi: 10.1371/journal.pone.0159912 (PMC4972430; doi:10.1371/journal.pone.0159912)

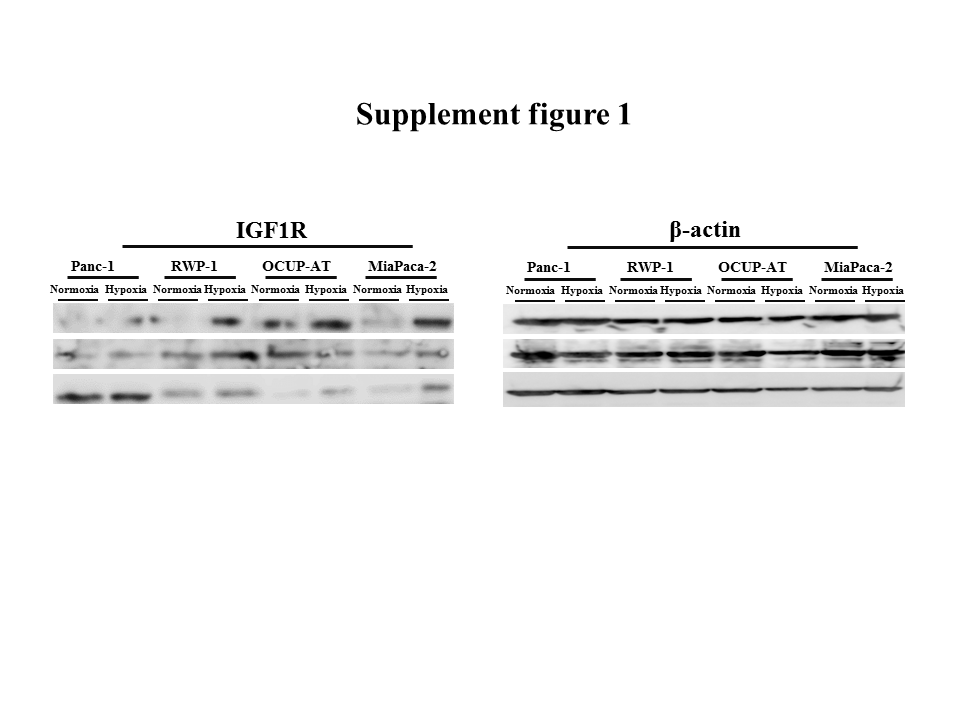

Supplement: S1 Fig — Three western pictures from 3 independent experiments were shown. (TIF) [file pone.0159912.s001.TIF]

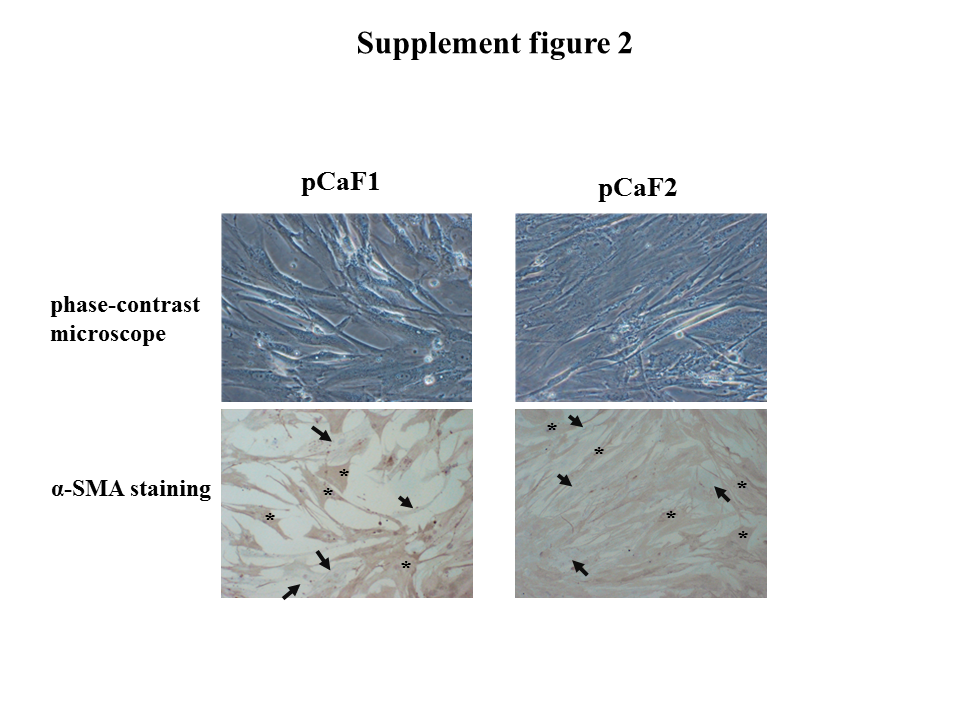

Supplement: S2 Fig — Both pCaF1 and pCaF2 cells contain αSMA-positive cells (asterisks) and αSMA-negative cells (arrows). (TIF) [file pone.0159912.s002.TIF]

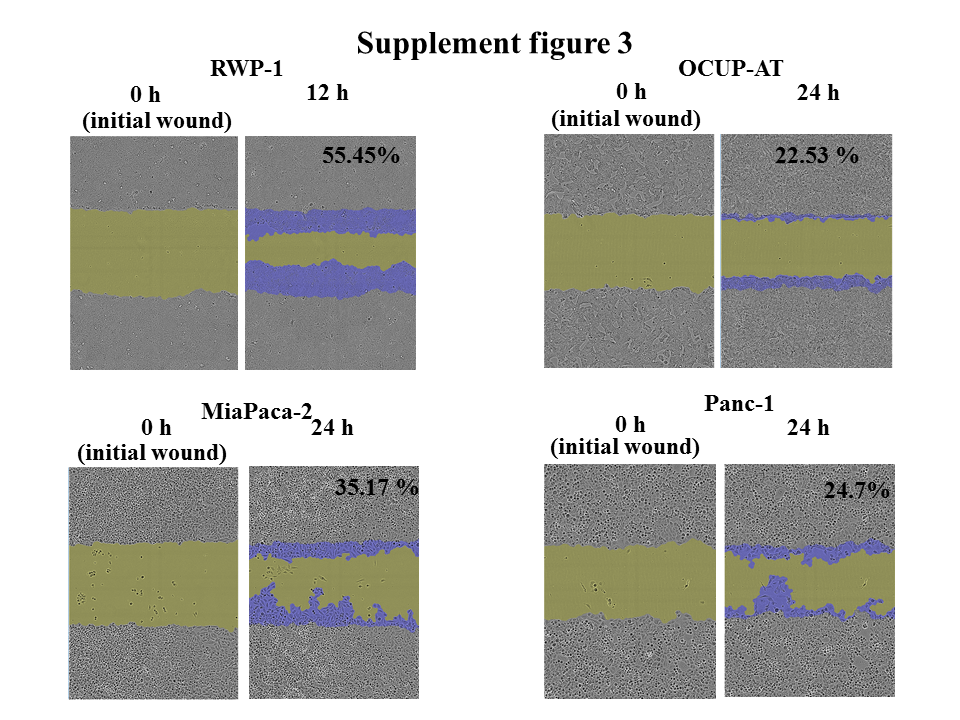

Supplement: S3 Fig — Pictures shows initial wound mask at 0 hours (yellow) and wound mask at 12 or 24 hours (blue). Relative wound confluence (%) was calculated as 100 x wound closure area at each time (blue) /wound area at time 0 (yellow). (TIF) [file pone.0159912.s003.TIF]
